# Methyl (*E*)-3,5-dimeth­oxy-2-{[2-(4-meth­oxy­benzo­yl)hydrazin-1-yl­idene]meth­yl}benzoate

**DOI:** 10.1107/S1600536812034782

**Published:** 2012-08-11

**Authors:** Humera Naz, Muhammad Taha, Aqilah Abd Rahman, Nor Hadiani Ismail, Sammer Yousuf

**Affiliations:** a Atta-ur-Rahman Research Institute for Natural Products Discovery (RiND), Universiti Tecknologi MARA, Puncak Alam 42300, Selangor, Malaysia; b Faculty of Pharmacy, Universiti Tecknologi MARA, Puncak Alam 42300, Selangor, Malaysia; c H.E.J. Research Institute of Chemistry, International Center for Chemical and Biological Sciences, University of Karachi, Karachi 75270, Pakistan

## Abstract

In the title compound, C_19_H_20_N_2_O_6_, the azomethine [C=N = 1.269 (2) Å] double bond adopts an *E* conformation and the dihedral angle between the planes of the benzene rings is 17.41 (11)°. In the crystal, inversion dimers linked by pairs of N—H⋯O hydrogen bonds generate *R*_2_^2^(16) loops. The dimers are connected by C—H⋯O and C—H⋯N hydrogen bonds, forming sheets lying parallel to (100).

## Related literature

For the biological activity of benzohydraazides, see: Khan *et al.* (2011 ▶); Chahan *et al.* (2006 ▶). For a related structure, see: Zhang (2009 ▶). 
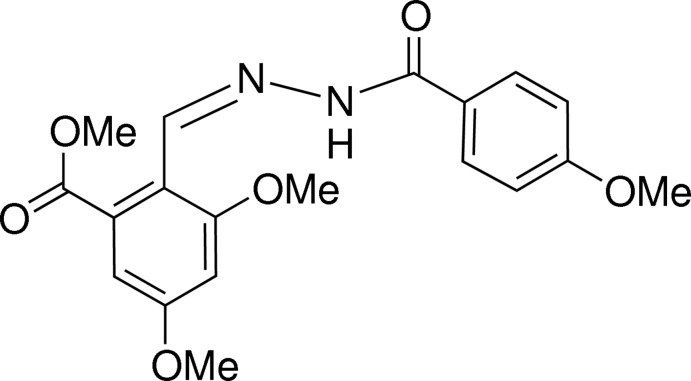


## Experimental

### 

#### Crystal data

C_19_H_20_N_2_O_6_
*M* *_r_* = 372.37
Triclinic, 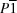

*a* = 8.8468 (7) Å
*b* = 10.7392 (8) Å
*c* = 10.9764 (8) Å
α = 113.377 (2)°
β = 90.656 (2)°
γ = 104.695 (2)°
*V* = 918.52 (12) Å^3^
*Z* = 2
Mo *K*α radiation
μ = 0.10 mm^−1^
*T* = 298 K
0.28 × 0.14 × 0.11 mm

#### Data collection

Bruker SMART APEX CCD diffractometer
Absorption correction: multi-scan (*SADABS*; Bruker, 2000 ▶) *T*_min_ = 0.972, *T*_max_ = 0.989
10426 measured reflections
3415 independent reflections
2224 reflections with *I* > 2σ(*I*)
*R*_int_ = 0.033

#### Refinement

*R*[*F* ^2^ > 2σ(*F* ^2^)] = 0.047
*wR*(*F* ^2^) = 0.114
*S* = 1.02
3415 reflections
252 parameters
H atoms treated by a mixture of independent and constrained refinement
Δρ_max_ = 0.16 e Å^−3^
Δρ_min_ = −0.17 e Å^−3^

### 

Data collection: *SMART* (Bruker, 2000 ▶); cell refinement: *SAINT* (Bruker, 2000 ▶); data reduction: *SAINT*; program(s) used to solve structure: *SHELXS97* (Sheldrick, 2008 ▶); program(s) used to refine structure: *SHELXL97* (Sheldrick, 2008 ▶); molecular graphics: *SHELXTL* (Sheldrick, 2008 ▶); software used to prepare material for publication: *SHELXTL*, *PARST* (Nardelli, 1995 ▶) and *PLATON* (Spek, 2009 ▶).

## Supplementary Material

Crystal structure: contains datablock(s) global, I. DOI: 10.1107/S1600536812034782/hb6920sup1.cif

Structure factors: contains datablock(s) I. DOI: 10.1107/S1600536812034782/hb6920Isup2.hkl

Supplementary material file. DOI: 10.1107/S1600536812034782/hb6920Isup3.cml

Additional supplementary materials:  crystallographic information; 3D view; checkCIF report

## Figures and Tables

**Table 1:** Hydrogen-bond geometry (Å, °)

*D*—H⋯*A*	*D*—H	H⋯*A*	*D*⋯*A*	*D*—H⋯*A*
N1—H1*A*⋯O5^i^	0.84 (2)	2.13 (2)	2.969 (2)	172.3 (19)
C18—H18*B*⋯N2^ii^	0.96	2.62	3.501 (3)	153
C19—H19*B*⋯O5^iii^	0.96	2.57	3.511 (3)	168

Symmetry codes: (i) 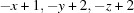; (ii) 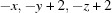; (iii) 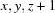.

**Fig. 1.**
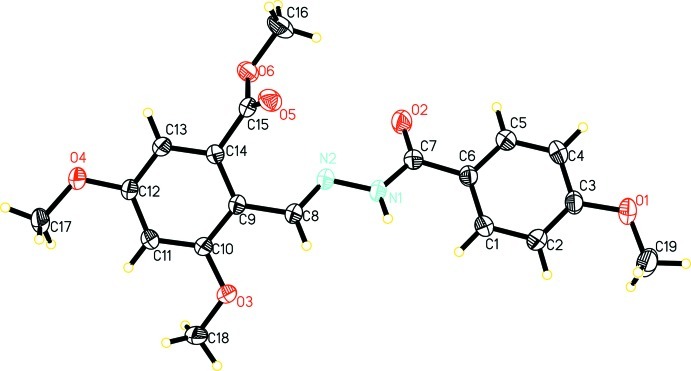
The molecular structure of (I) with displacement ellipsoids drawn at 30% probability level.

**Fig. 2.**
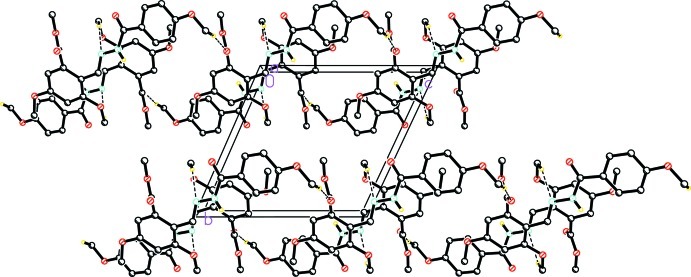
The crystal packing of the title compound I. Only hydrogen atoms involved in hydrogen bonding are shown.

## supplementary crystallographic information

### Comment

Phenyl hydrazones represent a very important class of bioactive organic compounds and are reported to have antibacterial, anticancer, antifungal, herbicidal activities, anticonvulsant, antiproliferative, antioxidant and antidiabetic activities (e.g. Khan *et al.*, 2011; Chahan *et al.*, 2006). The title compound was prepared as a part of our ongoing research to synthesize libraries of different bioactive benzohydrazone. The structure of title compound (Fig. 1) is similar to that of the previously published 4-Methoxy-*N*'-(2-methoxybenzylidene)-benzohydrazide (Zhang, 2009) with the difference that 2-methoxy benzne ring is replaced by 3,5-dimethoxybenzoate moiety (C9–C14). The azomethine (C=N,1.269 (2) Å) double bond adopt an *E* conformation (Fig. 1). The benzene rings (C1–C6 and C9–C14) subtend a dihedral angle 17.41 (11)° between them and maximum deviation of 0.014 (2) Å for C6 atom from the root mean square plane of 4-methoxybenzene ring (C1–C6).

The crystal structure features N1—H1A···O5, C18—H18B···N2 and C19—H19B···O5 intrmolecular hydrogen bonds and inked to form chains (symmetry codes as in Table 2) arranged parallel to (100) in (Fig. 2).

### Experimental

A mixture of 2 mmol of each 4-methoxybenzohydrazide and methyl 2-formyl-3,5-dimethoxybenzoate and catalytical amount of acetic acid was refluxed for 3 h. The progress of the reaction was monitored by TLC. After completion of reaction, the solvent was evaporated by vacuum to afford the crude product (0.610 g, yield 82%), which was re-crystallized from methanol solution to yield colourless blocks of the title compound.

### Refinement

H atoms on Methyl, phenyl and methine were positioned geometrically with C—H = 0.95 Å (CH_3_), and 0.93 Å (CH) and constrained to ride on their parent atoms with *U*_iso_(H)= 1.5*U*_eq_(CH_3_) 1.2*U*_eq_(CH). The H atoms on the nitrogen (N–H= 0.85 (2) Å) was located in difference Fourier maps and refined isotropically. A rotating group model was applied to the methyl groups.

### Crystal data

**Table:**

C_19_H_20_N_2_O_6_	*Z* = 2
*M**_r_* = 372.37	*F*(000) = 392
Triclinic, *P*1	*D*_x_ = 1.346 Mg m^−^^3^
*a* = 8.8468 (7) Å	Mo *K*α radiation, λ = 0.71073 Å
*b* = 10.7392 (8) Å	Cell parameters from 1645 reflections
*c* = 10.9764 (8) Å	θ = 2.0–25.5°
α = 113.377 (2)°	µ = 0.10 mm^−^^1^
β = 90.656 (2)°	*T* = 298 K
γ = 104.695 (2)°	Block, colorless
*V* = 918.52 (12) Å^3^	0.28 × 0.14 × 0.11 mm

### Data collection

**Table:**

Bruker SMART APEX CCD diffractometer	3415 independent reflections
Radiation source: fine-focus sealed tube	2224 reflections with *I* > 2σ(*I*)
Graphite monochromator	*R*_int_ = 0.033
ω scan	θ_max_ = 25.5°, θ_min_ = 2.0°
Absorption correction: multi-scan (*SADABS*; Bruker, 2000)	*h* = −10→10
*T*_min_ = 0.972, *T*_max_ = 0.989	*k* = −13→13
10426 measured reflections	*l* = −13→13

### Refinement

**Table:**

Refinement on *F*^2^	Primary atom site location: structure-invariant direct methods
Least-squares matrix: full	Secondary atom site location: difference Fourier map
*R*[*F*^2^ > 2σ(*F*^2^)] = 0.047	Hydrogen site location: inferred from neighbouring sites
*wR*(*F*^2^) = 0.114	H atoms treated by a mixture of independent and constrained refinement
*S* = 1.02	*w* = 1/[σ^2^(*F*_o_^2^) + (0.0494*P*)^2^ + 0.0289*P*] where *P* = (*F*_o_^2^ + 2*F*_c_^2^)/3
3415 reflections	(Δ/σ)_max_ < 0.001
252 parameters	Δρ_max_ = 0.16 e Å^−^^3^
0 restraints	Δρ_min_ = −0.17 e Å^−^^3^

### Special details

**Table:**

Geometry. All e.s.d.'s (except the e.s.d. in the dihedral angle between two l.s. planes) are estimated using the full covariance matrix. The cell e.s.d.'s are taken into account individually in the estimation of e.s.d.'s in distances, angles and torsion angles; correlations between e.s.d.'s in cell parameters are only used when they are defined by crystal symmetry. An approximate (isotropic) treatment of cell e.s.d.'s is used for estimating e.s.d.'s involving l.s. planes.
Refinement. Refinement of *F*^2^ against ALL reflections. The weighted *R*-factor *wR* and goodness of fit *S* are based on *F*^2^, conventional *R*-factors *R* are based on *F*, with *F* set to zero for negative *F*^2^. The threshold expression of *F*^2^ > σ(*F*^2^) is used only for calculating *R*-factors(gt) *etc*. and is not relevant to the choice of reflections for refinement. *R*-factors based on *F*^2^ are statistically about twice as large as those based on *F*, and *R*- factors based on ALL data will be even larger.

### Fractional atomic coordinates and isotropic or equivalent isotropic displacement parameters (Å^2^)

**Table:**

	*x*	*y*	*z*	*U*_iso_*/*U*_eq_	
O1	0.6563 (2)	0.64890 (18)	1.46257 (16)	0.0820 (5)	
O2	0.24378 (19)	0.62729 (15)	0.97724 (16)	0.0730 (5)	
O3	0.17303 (18)	1.25061 (14)	1.10340 (13)	0.0614 (4)	
O4	0.0119 (2)	1.17046 (16)	0.65074 (14)	0.0691 (5)	
O5	0.40868 (17)	0.87627 (14)	0.73902 (14)	0.0564 (4)	
O6	0.16475 (16)	0.73913 (14)	0.65091 (13)	0.0549 (4)	
N1	0.3446 (2)	0.86420 (19)	1.08462 (18)	0.0507 (5)	
N2	0.26850 (18)	0.88947 (17)	0.98968 (16)	0.0477 (4)	
C1	0.4660 (3)	0.8228 (2)	1.3057 (2)	0.0636 (6)	
H1B	0.4472	0.9098	1.3257	0.076*	
C2	0.5451 (3)	0.8037 (2)	1.4035 (2)	0.0631 (6)	
H2B	0.5770	0.8766	1.4884	0.076*	
C3	0.5760 (2)	0.6779 (2)	1.3750 (2)	0.0537 (5)	
C4	0.5221 (3)	0.5694 (2)	1.2506 (2)	0.0721 (7)	
H4A	0.5402	0.4823	1.2311	0.087*	
C5	0.4413 (3)	0.5890 (2)	1.1549 (2)	0.0603 (6)	
H5A	0.4043	0.5142	1.0717	0.072*	
C6	0.4143 (2)	0.7167 (2)	1.17971 (19)	0.0448 (5)	
C7	0.3274 (2)	0.7306 (2)	1.0710 (2)	0.0488 (5)	
C8	0.2808 (2)	1.0184 (2)	1.01728 (19)	0.0475 (5)	
H8A	0.3342	1.0878	1.0990	0.057*	
C9	0.2117 (2)	1.05887 (19)	0.92205 (18)	0.0410 (5)	
C10	0.1566 (2)	1.17821 (19)	0.96804 (18)	0.0434 (5)	
C11	0.0895 (2)	1.2190 (2)	0.88060 (19)	0.0491 (5)	
H11A	0.0554	1.3001	0.9129	0.059*	
C12	0.0739 (2)	1.1378 (2)	0.74496 (19)	0.0480 (5)	
C13	0.1234 (2)	1.0168 (2)	0.69618 (19)	0.0493 (5)	
H13A	0.1094	0.9611	0.6047	0.059*	
C14	0.1937 (2)	0.97907 (19)	0.78374 (18)	0.0419 (5)	
C15	0.2684 (3)	0.8614 (2)	0.72612 (18)	0.0442 (5)	
C16	0.2293 (3)	0.6196 (2)	0.5973 (3)	0.0761 (7)	
H16A	0.1461	0.5353	0.5470	0.114*	
H16B	0.2788	0.6089	0.6694	0.114*	
H16C	0.3058	0.6353	0.5398	0.114*	
C17	−0.0109 (3)	1.3070 (2)	0.6920 (2)	0.0695 (7)	
H17A	−0.0380	1.3215	0.6146	0.104*	
H17B	0.0844	1.3775	0.7420	0.104*	
H17C	−0.0945	1.3142	0.7471	0.104*	
C18	0.0967 (3)	1.3593 (2)	1.1573 (2)	0.0617 (6)	
H18A	0.1147	1.3988	1.2532	0.093*	
H18B	−0.0145	1.3203	1.1282	0.093*	
H18C	0.1383	1.4320	1.1268	0.093*	
C19	0.7245 (3)	0.7605 (3)	1.5882 (3)	0.0943 (9)	
H19A	0.7883	0.7292	1.6350	0.141*	
H19B	0.6425	0.7883	1.6400	0.141*	
H19C	0.7888	0.8396	1.5750	0.141*	
H1A	0.412 (2)	0.936 (2)	1.141 (2)	0.054 (7)*	

### Atomic displacement parameters (Å^2^)

**Table:**

	*U*^11^	*U*^22^	*U*^33^	*U*^12^	*U*^13^	*U*^23^
O1	0.1044 (13)	0.1002 (13)	0.0638 (11)	0.0587 (11)	−0.0016 (10)	0.0388 (10)
O2	0.0884 (12)	0.0517 (9)	0.0698 (11)	0.0157 (9)	−0.0233 (9)	0.0191 (8)
O3	0.0889 (11)	0.0613 (9)	0.0364 (8)	0.0409 (8)	−0.0007 (7)	0.0109 (7)
O4	0.1052 (12)	0.0728 (10)	0.0478 (9)	0.0528 (9)	0.0012 (8)	0.0271 (8)
O5	0.0507 (9)	0.0559 (9)	0.0602 (9)	0.0234 (7)	0.0022 (7)	0.0164 (7)
O6	0.0585 (9)	0.0437 (8)	0.0583 (9)	0.0197 (7)	−0.0020 (7)	0.0138 (7)
N1	0.0596 (12)	0.0468 (11)	0.0467 (11)	0.0137 (10)	−0.0095 (9)	0.0216 (9)
N2	0.0520 (10)	0.0511 (11)	0.0444 (10)	0.0180 (8)	−0.0025 (8)	0.0225 (8)
C1	0.0948 (18)	0.0525 (13)	0.0532 (14)	0.0362 (13)	0.0017 (13)	0.0225 (11)
C2	0.0890 (17)	0.0623 (15)	0.0427 (13)	0.0324 (13)	0.0019 (12)	0.0200 (11)
C3	0.0572 (13)	0.0682 (15)	0.0521 (13)	0.0309 (12)	0.0097 (11)	0.0333 (12)
C4	0.0968 (19)	0.0615 (15)	0.0710 (17)	0.0434 (14)	−0.0009 (15)	0.0279 (13)
C5	0.0732 (15)	0.0511 (13)	0.0551 (14)	0.0251 (12)	−0.0022 (12)	0.0162 (11)
C6	0.0472 (12)	0.0476 (12)	0.0461 (12)	0.0172 (10)	0.0074 (10)	0.0235 (10)
C7	0.0529 (13)	0.0457 (13)	0.0476 (12)	0.0164 (11)	0.0014 (10)	0.0177 (10)
C8	0.0559 (13)	0.0474 (12)	0.0402 (11)	0.0182 (10)	−0.0031 (9)	0.0171 (9)
C9	0.0432 (11)	0.0439 (11)	0.0380 (11)	0.0144 (9)	−0.0002 (9)	0.0180 (9)
C10	0.0508 (12)	0.0436 (11)	0.0360 (11)	0.0180 (10)	0.0012 (9)	0.0138 (9)
C11	0.0607 (13)	0.0476 (12)	0.0467 (12)	0.0279 (10)	0.0057 (10)	0.0197 (10)
C12	0.0583 (13)	0.0541 (13)	0.0407 (12)	0.0250 (10)	0.0026 (10)	0.0233 (10)
C13	0.0610 (13)	0.0531 (13)	0.0361 (11)	0.0259 (11)	0.0017 (10)	0.0149 (9)
C14	0.0460 (11)	0.0426 (11)	0.0410 (11)	0.0179 (9)	0.0034 (9)	0.0178 (9)
C15	0.0536 (13)	0.0476 (12)	0.0347 (11)	0.0175 (11)	0.0005 (10)	0.0184 (9)
C16	0.0824 (17)	0.0435 (13)	0.0919 (18)	0.0268 (12)	0.0028 (15)	0.0121 (12)
C17	0.0943 (18)	0.0737 (16)	0.0675 (16)	0.0477 (14)	0.0140 (14)	0.0416 (13)
C18	0.0781 (16)	0.0566 (14)	0.0459 (13)	0.0327 (12)	0.0067 (11)	0.0082 (10)
C19	0.106 (2)	0.133 (3)	0.0602 (17)	0.065 (2)	−0.0023 (16)	0.0375 (17)

### Geometric parameters (Å, º)

**Table:**

O1—C3	1.364 (2)	C6—C7	1.488 (3)
O1—C19	1.416 (3)	C8—C9	1.461 (2)
O2—C7	1.224 (2)	C8—H8A	0.9300
O3—C10	1.362 (2)	C9—C10	1.394 (2)
O3—C18	1.425 (2)	C9—C14	1.399 (3)
O4—C12	1.366 (2)	C10—C11	1.385 (2)
O4—C17	1.422 (2)	C11—C12	1.378 (3)
O5—C15	1.209 (2)	C11—H11A	0.9300
O6—C15	1.326 (2)	C12—C13	1.382 (3)
O6—C16	1.450 (2)	C13—C14	1.377 (2)
N1—C7	1.350 (2)	C13—H13A	0.9300
N1—N2	1.383 (2)	C14—C15	1.494 (3)
N1—H1A	0.85 (2)	C16—H16A	0.9600
N2—C8	1.269 (2)	C16—H16B	0.9600
C1—C6	1.375 (3)	C16—H16C	0.9600
C1—C2	1.384 (3)	C17—H17A	0.9600
C1—H1B	0.9300	C17—H17B	0.9600
C2—C3	1.361 (3)	C17—H17C	0.9600
C2—H2B	0.9300	C18—H18A	0.9600
C3—C4	1.375 (3)	C18—H18B	0.9600
C4—C5	1.376 (3)	C18—H18C	0.9600
C4—H4A	0.9300	C19—H19A	0.9600
C5—C6	1.373 (3)	C19—H19B	0.9600
C5—H5A	0.9300	C19—H19C	0.9600
			
C3—O1—C19	118.05 (19)	C12—C11—H11A	120.5
C10—O3—C18	118.26 (15)	C10—C11—H11A	120.5
C12—O4—C17	118.22 (16)	O4—C12—C11	123.46 (18)
C15—O6—C16	114.98 (16)	O4—C12—C13	115.72 (17)
C7—N1—N2	120.43 (18)	C11—C12—C13	120.82 (17)
C7—N1—H1A	124.2 (14)	C14—C13—C12	119.67 (18)
N2—N1—H1A	114.6 (14)	C14—C13—H13A	120.2
C8—N2—N1	115.63 (17)	C12—C13—H13A	120.2
C6—C1—C2	121.78 (19)	C13—C14—C9	121.17 (17)
C6—C1—H1B	119.1	C13—C14—C15	117.78 (17)
C2—C1—H1B	119.1	C9—C14—C15	120.59 (16)
C3—C2—C1	119.8 (2)	O5—C15—O6	123.04 (18)
C3—C2—H2B	120.1	O5—C15—C14	124.35 (18)
C1—C2—H2B	120.1	O6—C15—C14	112.46 (17)
C2—C3—O1	124.7 (2)	O6—C16—H16A	109.5
C2—C3—C4	119.3 (2)	O6—C16—H16B	109.5
O1—C3—C4	116.04 (19)	H16A—C16—H16B	109.5
C3—C4—C5	120.3 (2)	O6—C16—H16C	109.5
C3—C4—H4A	119.8	H16A—C16—H16C	109.5
C5—C4—H4A	119.8	H16B—C16—H16C	109.5
C6—C5—C4	121.4 (2)	O4—C17—H17A	109.5
C6—C5—H5A	119.3	O4—C17—H17B	109.5
C4—C5—H5A	119.3	H17A—C17—H17B	109.5
C5—C6—C1	117.35 (19)	O4—C17—H17C	109.5
C5—C6—C7	118.43 (18)	H17A—C17—H17C	109.5
C1—C6—C7	124.20 (18)	H17B—C17—H17C	109.5
O2—C7—N1	122.64 (19)	O3—C18—H18A	109.5
O2—C7—C6	121.79 (18)	O3—C18—H18B	109.5
N1—C7—C6	115.55 (18)	H18A—C18—H18B	109.5
N2—C8—C9	120.71 (18)	O3—C18—H18C	109.5
N2—C8—H8A	119.6	H18A—C18—H18C	109.5
C9—C8—H8A	119.6	H18B—C18—H18C	109.5
C10—C9—C14	117.63 (16)	O1—C19—H19A	109.5
C10—C9—C8	120.10 (17)	O1—C19—H19B	109.5
C14—C9—C8	122.24 (17)	H19A—C19—H19B	109.5
O3—C10—C11	122.77 (17)	O1—C19—H19C	109.5
O3—C10—C9	115.61 (16)	H19A—C19—H19C	109.5
C11—C10—C9	121.61 (17)	H19B—C19—H19C	109.5
C12—C11—C10	119.05 (18)		
			
C7—N1—N2—C8	174.00 (18)	C14—C9—C10—O3	178.40 (17)
C6—C1—C2—C3	1.2 (4)	C8—C9—C10—O3	0.3 (3)
C1—C2—C3—O1	178.3 (2)	C14—C9—C10—C11	−1.4 (3)
C1—C2—C3—C4	−2.7 (3)	C8—C9—C10—C11	−179.51 (18)
C19—O1—C3—C2	−5.8 (3)	O3—C10—C11—C12	−178.44 (18)
C19—O1—C3—C4	175.3 (2)	C9—C10—C11—C12	1.4 (3)
C2—C3—C4—C5	1.7 (4)	C17—O4—C12—C11	11.9 (3)
O1—C3—C4—C5	−179.3 (2)	C17—O4—C12—C13	−167.25 (19)
C3—C4—C5—C6	0.9 (4)	C10—C11—C12—O4	−178.79 (19)
C4—C5—C6—C1	−2.4 (3)	C10—C11—C12—C13	0.4 (3)
C4—C5—C6—C7	179.4 (2)	O4—C12—C13—C14	177.21 (18)
C2—C1—C6—C5	1.4 (3)	C11—C12—C13—C14	−2.0 (3)
C2—C1—C6—C7	179.4 (2)	C12—C13—C14—C9	1.9 (3)
N2—N1—C7—O2	−1.1 (3)	C12—C13—C14—C15	−170.38 (18)
N2—N1—C7—C6	−179.71 (16)	C10—C9—C14—C13	−0.2 (3)
C5—C6—C7—O2	20.4 (3)	C8—C9—C14—C13	177.79 (18)
C1—C6—C7—O2	−157.6 (2)	C10—C9—C14—C15	171.85 (18)
C5—C6—C7—N1	−160.94 (19)	C8—C9—C14—C15	−10.1 (3)
C1—C6—C7—N1	21.0 (3)	C16—O6—C15—O5	7.0 (3)
N1—N2—C8—C9	177.12 (17)	C16—O6—C15—C14	−177.25 (18)
N2—C8—C9—C10	148.9 (2)	C13—C14—C15—O5	111.9 (2)
N2—C8—C9—C14	−29.1 (3)	C9—C14—C15—O5	−60.4 (3)
C18—O3—C10—C11	9.9 (3)	C13—C14—C15—O6	−63.8 (2)
C18—O3—C10—C9	−169.98 (18)	C9—C14—C15—O6	123.87 (19)

### Hydrogen-bond geometry (Å, º)

**Table:**

*D*—H···*A*	*D*—H	H···*A*	*D*···*A*	*D*—H···*A*
N1—H1*A*···O5^i^	0.84 (2)	2.13 (2)	2.969 (2)	172.3 (19)
C18—H18*B*···N2^ii^	0.96	2.62	3.501 (3)	153
C19—H19*B*···O5^iii^	0.96	2.57	3.511 (3)	168

Symmetry codes: (i) −*x*+1, −*y*+2, −*z*+2; (ii) −*x*, −*y*+2, −*z*+2; (iii) *x*, *y*, *z*+1.
